# Effect of training and sudden detraining on the patellar tendon and its enthesis in rats

**DOI:** 10.1186/1471-2474-12-20

**Published:** 2011-01-19

**Authors:** Antonio Frizziero, Milena Fini, Francesca Salamanna, Arsenio Veicsteinas, Nicola Maffulli, Marina Marini

**Affiliations:** 1 Department of Histology, Embryology and Applied Biology, University of Bologna, Italy; 2 Laboratory of Pre-clinic and Surgical Studies, Rizzoli Orthopaedic Institute, Bologna, Italy; 3 Department of Sport Science, Nutrition and Health, University of Milan, and Center of Sport Medicine, Don Gnocchi Foundation, Milan, Italy; 4 Queen Mary University of London, Barts and The London School of Medicine and Dentistry, Center for Sports and Exercise Medicine London, United Kingdom

## Abstract

**Background:**

Different conditions may alter tendon characteristics. Clinical evidence suggests that tendon injuries are more frequent in athletes that change type, intensity and duration of training. Aim of the study was the assessment of training and especially detraining on the patellar tendon (PT) and its enthesis.

**Methods:**

27 male adult Sprague-Dawley rats were divided into 3 groups: 20 rats were trained on a treadmill for 10 weeks. Of these, 10 rats were euthanized immediately after training (trained group), and 10 were caged without exercise for 4 weeks before being euthanized (de-trained group). The remaining 7 rats were used as controls (untrained rats). PT insertion, structure (collagen fiber organization and proteoglycan, PG, content), PT thickness, enthesis area, and subchondral bone volume at the enthesis were measured by histomorphometry and microtomography.

**Results:**

Both PG content and collagen fiber organization were significantly lower in untrained and detrained animals than in trained ones (*p *< 0.05 and *p *< 0.0001). In the detrained group, fiber organization and PG content were worse than that of the untrained groups and the untrained group showed a significantly higher score than the detrained group (*p *< 0.05). In the trained group, the PT was significantly thicker than in untrained group (*p *< 0.05). No significant differences in the enthesis area and subchondral bone volume among the three groups were seen.

**Conclusions:**

Moderate exercise exerts a protective effect on the PT structure while sudden discontinuation of physical activity has a negative effect on tendons. The present results suggest that after a period of sudden de-training (such as after an injury) physical activity should be restarted with caution and with appropriate rehabilitation programs.

## Background

Tendon is a highly organized connective tissue, capable of resisting high tensile strength while transmitting forces from muscle to bone. The region where a tendon, ligament or joint capsule attaches to bone is the enthesis. In the context of a tendon, the enthesis is a fibrous or fibrocartilagineous zone of tendon attachment to bone that ensures that the contractile forces produced by the muscle contraction are transmitted to the skeleton [1].

Tendons consist of a densely packed well organized connective tissue dominated by collagen (60-85% of tendon dry weight and mainly collagen I) arranged into fibrils, fibers, bundles, and fascicles, embedded in extracellular matrix (ECM) proteins, including proteoglycans (PG). Mechanical stimuli have different impact on the expression of PG in tendons [2-6]. For example, mechanical tension induces the synthesis of decorin, whereas the production of the large PG aggrecan is stimulated by compression [7].

A variety of conditions, including immobilization, training, aging and medications, may influence tendon characteristics [2,8-11]. Immobilization results in profound reduction in the mechanical properties of tendon. Most noticeably, there is a decrease in tendon strength and an increase in collagen turnover [12]. With exercise, the turnover of mature collagen and collagen cross-links increases [13,14], large diameter fibrils are formed with increased packing density of fibrils [15] and increased tendon stiffness [16]. Exercise also leads to changes in PG content [17]. Strenuous exercise in mature rodents or chickens leads to thickening of collagen fibrils and increase in the galactosamine-containing glycosaminoglycans (GAGs) [18]. In contrast, immature tendons respond to exercise with higher collagen turnover, reduced maturation of collagen [19] and changes in hyaluronan concentration [20]. Kubo et al observed that tendon adaptation to detraining is faster than the one to resistance training [21], but to the present author knowledge, in comparison with training or immobilization, the effects of sudden detraining on tendons have not been deeply investigated.

In fact, there is a paucity of data examining the impact that cessation of training may have on tendon. In practice, we do not fully understand how tendons respond to a period of training followed by sudden detraining. On the other site, clinical evidence suggests that tendon injuries are more frequent in athletes that change type, intensity and duration of training. Thus, the aim of the present research was the study of PT and its enthesis after training and especially following sudden discontinuation of activity. We evaluated the histological characteristics of the patellar tendon (PT) and its enthesis in trained, detrained, and untrained rats. Because both the magnitude and the type of load transmission across the joint surface are likely to change with exercise and with tendon health status we measured subchondral bone thickness, bone area and bone volume. Also bone volume, as an indicator of bone adaption, was evaluated by microtomography at the level of the subchondral bone connected to the enthesis.

## Methods

### Animal and training protocol

The animal study was performed according to the Italian Law by Decree on Animal Experimentation (N. 116/92), and was approved by the Health Authority of Milan (Italy) Ethical Committee for the Use of Laboratory Animals. Training was carried out according to the American Physiological Society guidelines for exercising rodents on treadmills [22].

A total of 27 male albino Sprague-Dawley rats aged 9 weeks, between 250 g to 350 g, were placed in individual standard cages and fed a standard diet without limitations; room temperature was kept at 21 ± 2°C; 12 hrs of light were automatically alternated with 12 hrs of dark. After 1 week of acclimatization, 20 rats were randomly chosen to run on a six-lane rodent treadmill 1 hr a day, three times a week. The speed was gradually increased to reach 25 m/min in 5 weeks, which corresponds to ~ 60% VO2max, and was then maintained constant for a further 5 weeks [20]. Seven control untrained animals were placed on a non-moving treadmill during the training session and were euthanized at 19 weeks of age (untrained group). At the end of the 10-week training, under general anesthesia 10 trained rats were randomly chosen for immediate euthanasia (trained group) whereas 10 were caged without exercise for a further 4 weeks before being euthanized (detrained group).

The rats were periodically examined by a veterinarian. Food consumption and body weight were evaluated three times a week.

### Histological studies

The right and left knee joints were explanted and fixed in 4% buffered paraformaldehyde for 48 hours for un-decalcified bone processing. The samples were then dehydrated by placing them in graded series of increasing percentage of alcohol with one step in 50% alcohol, one step in 75% alcohol, two steps in 95% alcohol, and two steps in 100% alcohol, for 48 hours per each step. Finally, they were embedded in polymethyl-methacrylate (Merck, Shuchardt, Hohenbrunn, Germany). Blocks were sectioned in the sagittal plane. A series of sections 200 ± 100 μm thick, spaced about 300 μm apart because of the thickness of the microtome diamond saw, were obtained with a Leica 1600 diamond saw microtome (Leica SpA, Milan, Italy). The sections were then automatically thinned (EXAKT Cutting and Grinding Systems, GmbH & Co., Norderstedt, Germany) to 40 ± 10 μm with different abrasive papers (EXAKT Abrasive Disc, GmbH & Co., Norderstedt, Germany), from 1200 to 300 grit in steps of 15 minutes each. A total of 27 samples (10 from trained, 10 from detrained, and 7 from control animals) were processed and analyzed. They were stained with Toluidine Blu, Acid Fuchsin, Fast Green, and processed for routine histological and histomorphometrical analyses by using a transmission and polarized light AxioSkop Microscope (Carl Zeiss GmbH, Jena, Germany). Computerized image analysis was performed with Axio Vision 4.6 (Carl Zeiss GmbH, Jena, Germany). Three sections per each sample were analyzed by two blinded investigators.

### Histology and Histomorphometry

After light microscope evaluation, histomorphometric analyses were performed for the assessment of the structure and morphology of the PT. This evaluation was performed by using a semi-quantitative score which evaluated four parameters, according to Table 1, and measuring the enthesis area and the PT thickness. The PT insertion site was evaluated by examining tendon, fibrocartilage, mineralized fibrocartilage and bone at the insertion level at 10× magnification. The scores for this evaluation was 0, for normal insertion site and 1 for altered insertion site. Insertion site was considered as normal when collagen fiber organization continued into fibrocartilaginous and mineralized zones and bone beneath tendon was normal [23]. For the evaluation of PT metachromasia, the score evaluated the intensity of staining by Toluidine Blue O. This metachromatic dye binds to the ECM proteoglycans (PGs) staining the PT matrix blue [24]; "blue" was defined, on the red-green-blue (RGB) scale [25,26], as (R = 24; G = 15; B = 70; hexadecimal RGB value = 180F469). The analyses were carried out at 10× magnification. The method provides discrete numbers to which we assign the following scores: 0 for unstained tendons (70 ± 10% of pixels having hexadecimal RGB value = DAC3B1: R = 218; G = 195; B = 177), 1 for slightly stained tendons (70 ± 10% of pixels having hexadecimal RGB value = 8E5671: R = 142; G = 86; B = 113), 2 for partially and inhomogeneously stained tendons (70 ± 10% of pixels having hexadecimal RGB value = 5C426F: R = 92; G = 66; B = 111) and 3 for tendons with total and homogeneous staining (70 ± 10% of pixels having hexadecimal RGB value = 180F46: R = 24; G = 15; B = 70) (Table 1).

**Table 1:** Semi-quantitative score for the evaluation of patellar tendon

Parameter (enlargement)	Score	Meaning	RGB
Patellar tendon insertion site (10×)	0	Normal	
	1	Altered	
Patellar tendon methacromasia (10×)	0	Not stained	DAC3B1
	1	Slightly stained	8E5671
	2	Partially and unhomogenously stained	5C426F
	3	Total and homogeneously stained	180F46
Patellar tendon fiber organization/alignment (10×)	0	Completely disorganized	
	1	Partially disorganized	
	2	Slightly disorganized	
	3	Well organized	

Another parameter that was evaluated was the collagen PT fiber organization and alignment [23]. In normal conditions, these fibers are arranged in closely aligned bundles. The analysis was carried out at 10× magnification. The score for the fiber bundle evaluation was 0 for a complete disorganization of the fibers, with bundles lacking any alignment through their whole length, 1 for partial disorganization, with bundles non-aligned in more than half of the tendon length, 2 for slight disorganization, with bundles non-aligned in less than half of the tendon length and 3 for tendons with closely aligned bundles through their whole length. The score received by each sample was then averaged within the experimental group (trained, detrained and untrained), so that each group was assigned a total score between 0 and 7, with 0 corresponding to altered insertion site, unstained and not organized tendon, and 7 corresponding to a normal insertion site, stained and structurally well organized tendon.

Enthesis area (μm^2^) and PT thickness (μm) were also measured. The enthesis area was evaluated at a magnification of 10×. PT thickness was measured at a magnification of 2.5×, with 10 measurements performed along the length of the tendon. To avoid biases due to subjectivity, the histological evaluations and measurements were performed by two experienced and blinded investigators. Possible discrepancies were resolved by sharing the final results.

### Microcomputer Tomography (Micro-CT) Analyses

**Figure 1:**
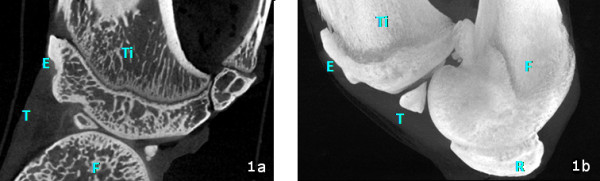
**Longitudinal plane of knee joint, Micro-CT image**: 1a) Microtomographic cross-section of knee joint; 1b) MIP (maximal intensity projection) of knee joint. T: patellar tendon; E: enthesis; Ti: tibia; F: femur; P: patella.

After dehydration in graded series of alcohols and before embedding in poly-methyl-methacrylate, 4 samples for each group were randomly chosen for analysis by Micro-CT SkyScan 1172 (μCT, SKYSCAN, Kartuizersweg 3B 2550 Kontich, Belgium). The system consisted of a micro focus X-ray source, a routable specimen holder and a detector system equipped with a 4000 × 2624 pixel CCD camera. The samples were wrapped in parafilm, to prevent drying, and scanned. Each X-ray projection was acquired in a 12-bit gray level image, 2000×1150 pixel, 10.97 μm image pixel sizes, with an aluminum filter and for a time of 35 minutes. After acquisition, projection images, 935 for the entire sample, were reconstructed (NRecon 1.4.4), and all the sections were cut along a longitudinal plane (CTAn 1.7) (Figure 1a,b). Then, a region of interest (ROI) of subchondral bone at the level of the enthesis was selected within the volume of the bone area where the PT inserts. This step was repeated for different sections arranged along the volume of the bone, and subsequently the ROI was interpolated. Bone volume and bone area were measured. To compare the same number of sections for each sample, the ROI dataset of every examined sample was reduced to the 70 most central sections. Subsequently, the images inside the selected ROI were binarized considering the bone as white, and the quantitative 2D and 3D parameters were analyzed.

### Statistical Analysis

Statistical evaluation was performed using the software package SPSS/PC+ Statistics TM 12.1 (SPSS Inc., Chicago, IL USA). After verifying the normal distribution of data, one-way ANOVA followed by the Scheffé *post hoc *multi comparison tests was used to analyze histomorphometric differences between groups.

## Results

### Histology and Histomorphometry

Histomorphometry data are reported in Table 2. In all groups, the PT tendon insertion was normal with collagen fiber organization continued into fibrocartilaginous and mineralized zones with healthy bone beneath the tendon. The untrained group yielded a total score of 3.10 ± 0.53, and PT displayed the most reduced staining, although, remarkably, one single untrained control scored 3. The total score was 4.86 ± 0.53 for the trained group and 2.33 ± 0.36 for the detrained group, respectively. Comparison ANOVA showed that in the trained group, the total score was significantly higher than in the detrained and in the untrained groups (*p *< 0.0005). The untrained group showed a significantly higher score than the detrained group (*p *< 0.05).

**Table 2:** Histomorphometric scores of PT in Untrained, Trained and Detrained rats

Group	Score	Tendon thickness (μm)
	***Mean ± SD***	***Mean ± SD***
**Untrained**		**538 ± 155**
PT insertion site	1	
PT methacromasia	0.5 ± 1.03	
PT fiber organization/alignement	1.75 ± 1.18	
**TOTAL**	3.10 ± 0.53*	
**Trained**		**807 ± 138^a^**
PT insertion site	1	
PT methacromasia	1.25 ± 0.68 ^a, b^	
PT fiber organization/alignement	2.87 ± 0.44 ^a, c^	
**TOTAL**	4.86 ± 0.53**	
**Detrained**		**612 ± 170**
PT insertion site	1	
PT methacromasia	0.4 ± 0.51	
PT fiber organization/alignement	0.9 ± 1.19	
**TOTAL**	**2.33 ± 0.36**	

* *p *< 0.05 (untrained *vs *detrained); ** *p *< 0.0005 (trained *vs *detrained; trained *vs *untrained).

^a ^*p *< 0.05 (trained *vs *untrained);^b ^*p *< 0.05 (trained *vs *detrained); ^c ^*p *< 0.0001 (trained *vs *detrained)

More in detail, histomorphometry showed that both PG content and collagen fiber organization were significantly lower in untrained and detrained animals than in trained ones (*p *< 0.05 and *p *< 0.0001).

**Figure 2:**
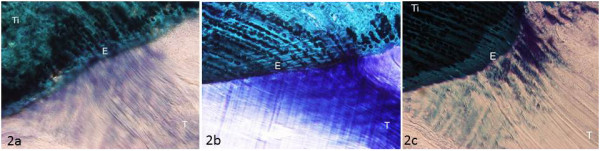
**Toluidine-blue, Acid Fucsin, Fast Green stained sections of patellar tendon**: 2a) Untrained group: patellar tendon with partially disorganized collagen bundles; 2b) Trained group: patellar tendon with collagen fibers in parallel arrays, closely packed together; 2c) Detrained group: patellar tendon with partially disorganized collagen bundles. Ti: tibia; T: patellar tendon; E: enthesis. Magnification 20×.

Collagen fibers were partially disorganized in untrained rat tendons (mean score: 1.75 ± 1.18) (Figure 2a). On the other hand, the samples of the trained group displayed closely aligned bundles; collagen fibers were parallel and closely packed together and with PGs neatly arranged (mean score: 2.87 ± 0.44) (Figure 2b). Therefore, 9 tendons of the trained group received a score of 3, and one sample scored 1. In the detrained group, fiber organization was worse than that of both the trained and the untrained groups, with disorganization of the collagen bundles (Figure 2c); 9 tendons scored 0-1 and only one sample which scored 3 (mean score: 0.9 ± 1.19).

**Figure 3:**
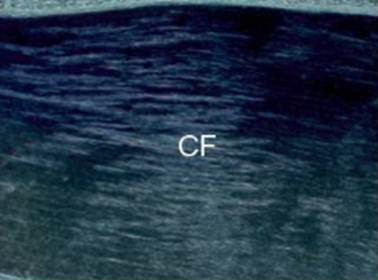
**Fibril birefringence observed with polarized microscopy in patellar tendon from the trained group**. CF: collagen fibers. Magnification 10×.

Polarized microscopy, showed that even the smallest visible collagen fibril was birifrangent, which indicates the presence of elongated submicroscopic units oriented in the direction of the fiber axis. Such characteristic is particularly conspicuous in the trained tendon (Figure 3).

**Figure 4:**
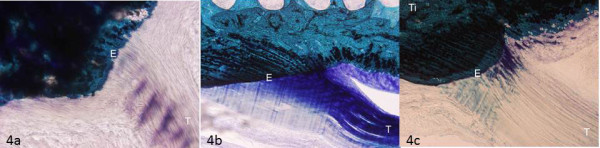
**Toluidine-blue, Acid Fucsin, Fast Green stained sections of patellar tendon showing collagen fibers methacromasia in the longitudinal plane of the knee joint**: 4a) Untrained group: collagen fibers of the patellar tendon displaying slight staining; 4b) Trained group: collagen fibers of the patellar tendon displaying homogeneous staining; 4c) Detrained group: patellar tendon collagen fibers devoid of staining. Ti: tibia; T: patellar tendon; E: enthesis. Magnification 10×.

In the untrained and detrained groups, collagen fibers slightly stained with toluidine blue O (means score: 0.5 ± 1.03 and 0.4 ± 0.51, respectively), while in the trained group collagen fibers were homogeneously stained and therefore with a physiologic amount of PGs (mean score: 1.25 ± 0.68) (Figure 4a,b,c).

Histomorphometric measurements showed no significant differences in the enthesis area among the three groups (untrained group: 2.7 ± 0.9 μm^2^; trained group: 2.3 ± 0.4 μm^2^; detrained group: 2.6 ± 0.5 μm^2^). In the trained group, the PT was significantly thicker than in untrained group (*p *< 0.05), while no significant differences were observed between the trained and detrained groups, and detrained and untrained groups (Table 2).

### Microtomography

Microtomographic analysis showed no significant differences in subchondral bone volume among the three groups (untrained group: 3.08 ± 0.2 mm^3^; trained group: 2.37 ± 0.15 mm^3^; detrained group: 2.72 ± 0.3 mm^3^).

## Discussion

Tendon is not a static tissue, rather it adapts itself according to the level, direction and frequency of the applied load through a process of remodeling probably mediated by tenocytes [27,28].

It is well known that running activity improves PT and its enthesis [29,30]. On the contrary there is a paucity of data examining the impact that a sudden detraining may have on tendons.

We studied several structural features of the PT in trained, de-trained and control untrained rats by histological, histomorphometric and microtomographic analyses using a semi-quantitative score which took into account several aspects of tendon morphology/structure and by measuring enthesis area, PT thickness and subchondral bone volume and collagen fiber arrangement and stainability. On the other hand, it was not possible to evaluate other parameters, since, in order to evaluate the enthesis area and to maintain the integrity at the tendon-bone interface, the joints were embedded in methacrylate and then processed for non decalcified histology (without undergoing the decalcification procedure). Therefore, this procedure did not allow the use of some quantitative analyses and the adoption of commonly used scores for the semiquantitative evaluation of tendinopathy, as those suggested by Movin and Bonar [31]. Thus it was not possible to measure cellularity, cellular activity, biochemical properties and vascularity of tendon.

Tendon thickness was also measured because this parameter changes with mechanical loading [32].

The training-induced modifications that we observed in the PT are in agreement with published data showing that exercise increases mature collagen replacement and fibril diameter and density. It has been also described that in exercising rodents the diameter of collagen fibrils increases, together with the galactosamine content in GAGs [16,18]. Exercise modifies PG content, as evidenced by Toluidine Blue staining of calcified sections [16]. Moreover, tendon thickness changes with mechanical loading [32]. These modifications may be associated with moderate rather than intense exercise and may have a protective effect [7,10]. In fact, *in vitro *repetitive application of high tensile stresses to tenocytes stimulates collagen synthesis, pro-inflammatory cytokines and gene expression of mediators, such as cyclooxygenase 2 (Cox-2), prostaglandin E2 (PGE2), matrix metalloproteinase 1 (MMP-1) [32,33]. Low tensile stresses have the opposite effects, with a reduction in pro-inflammatory mediators. It is still unclear how these findings can be applied to the management of tendinopathy [34,35]. Tensile and compressive forces exert a different influence on the expression of PGs in tendons. Tension induces decorin syntheses, and compression stimulates the production of aggrecan [9,11].

In detrained rats, fiber re-arrangement might arise from a sudden interruption of training which induces a decrease in the synthetic activities of the tenocytes. A sudden build-up of MMPs associated with training interruption might be also involved. In fact, it may be advanced the hypothesis that after the cessation of physical activity, PG synthesis is reduced, while MMPs, that had been synthesized during the training phase to help the fiber remodeling processes, persist and contribute to the disruption of bundle organization.

Subchondral bone undergoes functional adaptation in response to exercise, because both the magnitude and the type of load across the joint surface are likely to change with exercise. Casting immobilization and exercise are accompanied by remodeling of bone at the insertion of tendon and ligament even if it is still unclear if or how this process alters the enthesis [28]. Therefore, training was expected to alter bone remodeling, quantity and mechanical properties [34,35]. In our study, no significant differences in the enthesis area and adjacent subchondral bone volume were observed among the groups. It is possible that lack to significant differences is due to the magnitude and type of load. In fact, in this study, rats did not undergo high intensity training, but only moderate exercise which did not seem to affect subchondral bone volume at the enthesis. On the other hand, training significantly increased PT thickness in trained rats compared to sedentary untrained ones. Moderate physical activity might have a protective effect on tendon structures, however, a sudden interruption of such activity may, at least in the short term, influence negatively tendon morphology.

The observed negative effect of sudden detraining on PT requires further investigation also because of the limitations of the present study and the novelty of the observations.

## Conclusions

Moderate prolonged physical activity exerts a protective effect on the tendon structure and morphology and induces an increase of PGs. Discontinuing such activity has the opposite effects, and, in the short term, disrupts intra-tendinous tendon morphology.

The present results suggest that after a period of sudden de-training (such as after an injury) physical activity should be restarted with caution and with appropriate rehabilitation programs because cessation of activity cause modifications of PT collagen organization and PG content.

## List of abbreviations

PT: Patellar tendon; PGs: proteoglycans; ECM: extracellular matrix; GAGs: glycosaminoglycans; Cox-2: cyclooxygenase 2; PGE2: prostaglandin E2; MMP-1: matrix metalloproteinase 1; MMPs: matrix metalloproteinases; RGB: hexadecimal values of Red, Green, Blue.

## Competing interests

The authors declare that they have no competing interests.

## Authors' contributions

AF has conceived the study, has participated in its design and was involved in drafting the manuscript. MF participated in the design of the study, performed the statistical and microtomographical analysis and was involved in drafting the manuscript. FS carried out the histological and histomorphometrical measures and was involved in drafting the manuscript. AV performed the animal study and was involved in drafting the manuscript. NM was involved in discussing the histological and histomorphometrical analyses and participated in drafting the manuscript. MM developed the experimental model design and the animal training and participated in drafting the manuscript. All authors read and approved the final manuscript.

## Pre-publication history

The pre-publication history for this paper can be accessed here:

http://www.biomedcentral.com/1471-2474/12/20/prepub
